# Development of a metabolites risk score for one-year mortality risk prediction in pancreatic adenocarcinoma patients

**DOI:** 10.18632/oncotarget.7108

**Published:** 2016-02-01

**Authors:** Andrea Fontana, Massimiliano Copetti, Iole Maria Di Gangi, Tommaso Mazza, Francesca Tavano, Domenica Gioffreda, Fulvio Mattivi, Angelo Andriulli, Urska Vrhovsek, Valerio Pazienza

**Affiliations:** ^1^ Unit of Biostatistics I.R.C.C.S. “Casa Sollievo della Sofferenza” Hospital, San Giovanni Rotondo (FG), Italy; ^2^ Department of Food Quality and Nutrition, Research and Innovation Centre, Fondazione Edmund Mach (FEM), San Michele all'Adige, Italy; ^3^ Unit of Bioinformatics, I.R.C.C.S. “Casa Sollievo della Sofferenza” Hospital, San Giovanni Rotondo (FG), Italy; ^4^ Gastroenterology Unit, I.R.C.C.S. “Casa Sollievo della Sofferenza” Hospital, San Giovanni Rotondo (FG), Italy

**Keywords:** pancreatic cancer, multiple risk score

## Abstract

**Purpose:**

Survival among patients with adenocarcinoma pancreatic cancer (PDCA) is highly variable, which ranges from 0% to 20% at 5 years. Such a wide range is due to tumor size and stage, as well other patients' characteristics. We analyzed alterations in the metabolomic profile, of PDCA patients, which are potentially predictive of patient's one-year mortality.

**Experimental design:**

A targeted metabolomic assay was conducted on serum samples of patients diagnosed with pancreatic cancer. Statistical analyses were performed only for those 27 patients with information on vital status at follow-up and baseline clinical features. Random Forest analysis was performed to identify all metabolites and clinical variables with the best capability to predict patient's mortality risk at one year. Regression coefficients were estimated from multivariable Weibull survival model, which included the most associated metabolites. Such coefficients were used as weights to build a metabolite risk score (MRS) which ranged from 0 (lowest mortality risk) to 1 (highest mortality risk). The stability of these weights were evaluated performing 10,000 bootstrap resamplings.

**Results:**

MRS was built as a weighted linear combination of the following five metabolites: Valine (HR = 0.62, 95%CI: 0.11–1.71 for each standard deviation (SD) of 98.57), Sphingomyeline C24:1 (HR = 2.66, 95%CI: 1.30–21.09, for each SD of 20.67), Lysine (HR = 0.36, 95%CI: 0.03–0.77, for each SD of 51.73), Tripentadecanoate TG15 (HR = 0.25, 95%CI: 0.01–0.82, for each SD of 2.88) and Symmetric dimethylarginine (HR = 2.24, 95%CI: 1.28–103.08, for each SD of 0.62), achieving a very high discrimination ability (survival c-statistic of 0.855, 95%CI: 0.816–0.894). Such association was still present even after adjusting for the most associated clinical variables (confounders).

**Conclusions:**

The mass spectrometry-based metabolomic profiling of serum represents a valid tool for discovering novel candidate biomarkers with prognostic ability to predict one-year mortality risk in patients with pancreatic adenocarcinoma.

## INTRODUCTION

Pancreatic cancer (PC) is a highly aggressive and chemoresistant cancer [1].

Recently, scientists are struggling to find out a biomarker which may highlight the prediction of an uprising PC.

Up to date several clinical serum markers for PC were proposed: *a)* carbohydrate antigen 19-9 (CA 19-9) which is the most commonly utilized, *b)* cell surface associated mucin (MUC1), *c)* carcinoembryonic antigen-related cell adhesion protein molecule 1 (CEACAM1), and more recently *d)* a pyruvate kinase variant (M2-PK) [2]. However, these markers lack sensitivity and specificity, as they are unfrequently elevated in the early stage of the cancerogenesis, and may be over-expressed also in various inflammatory conditions [2, 3]. On the other hand, scientists focused their attention also on investigating factors potentially involved into survival and/or therapy response. A number of genes were proven to be associated with survival and/or therapy outcome prediction. For instance, higher DNA methyltransferase 3B (DNMT3B) mRNA levels predict longer survival in PC patients in the presence of non-invasive tumor whereas higher DNMT3B mRNA levels were associated with a poor prognosis in the presence of invasive tumor [4]. Moreover human ribonucleotidereductase (RRM1), involved in the homeostasis of nucleotides pools affecting cell proliferation, migration and metastasis [5] was found to improve survival in gemcitabine-treated patients [6–9]. Human equilibrative nucleoside transporter 1 (hENT1) a drug transporter together with deoxycytidine kinase (DCK), a key enzyme that activates gemcitabine by phosphorylation, was also found to be associated with acquired resistance to gemcitabine both *in vitro* [10–12] and *in vivo* [13–17]. As several studies reported conflicting results, the finding of a potential biomarker which can early predict the uprising of the pancreatic cancer and/or the chemotherapy outcome still remain unsolved [18].

Herein we analyzed potential changes in the concentration levels of metabolites in serum samples of pancreatic cancer patients in order to find possible associations between the mutual concentration levels of these metabolites and clinical pathological features in a selected and well characterized cohort of patients with PDAC. Moreover, we built and described a multiple risk score (MRS) formula, as a weighted linear combination of all those metabolites which strongly predict one-year patient's mortality risk. The implementation of our MRS formula may help prioritize the use of available resources for targeting aggressive preventive and treatment strategies in a subset of very high-risk individuals.

## RESULTS

### Metabolites concentration levels in PC patients

**Figure 1:**
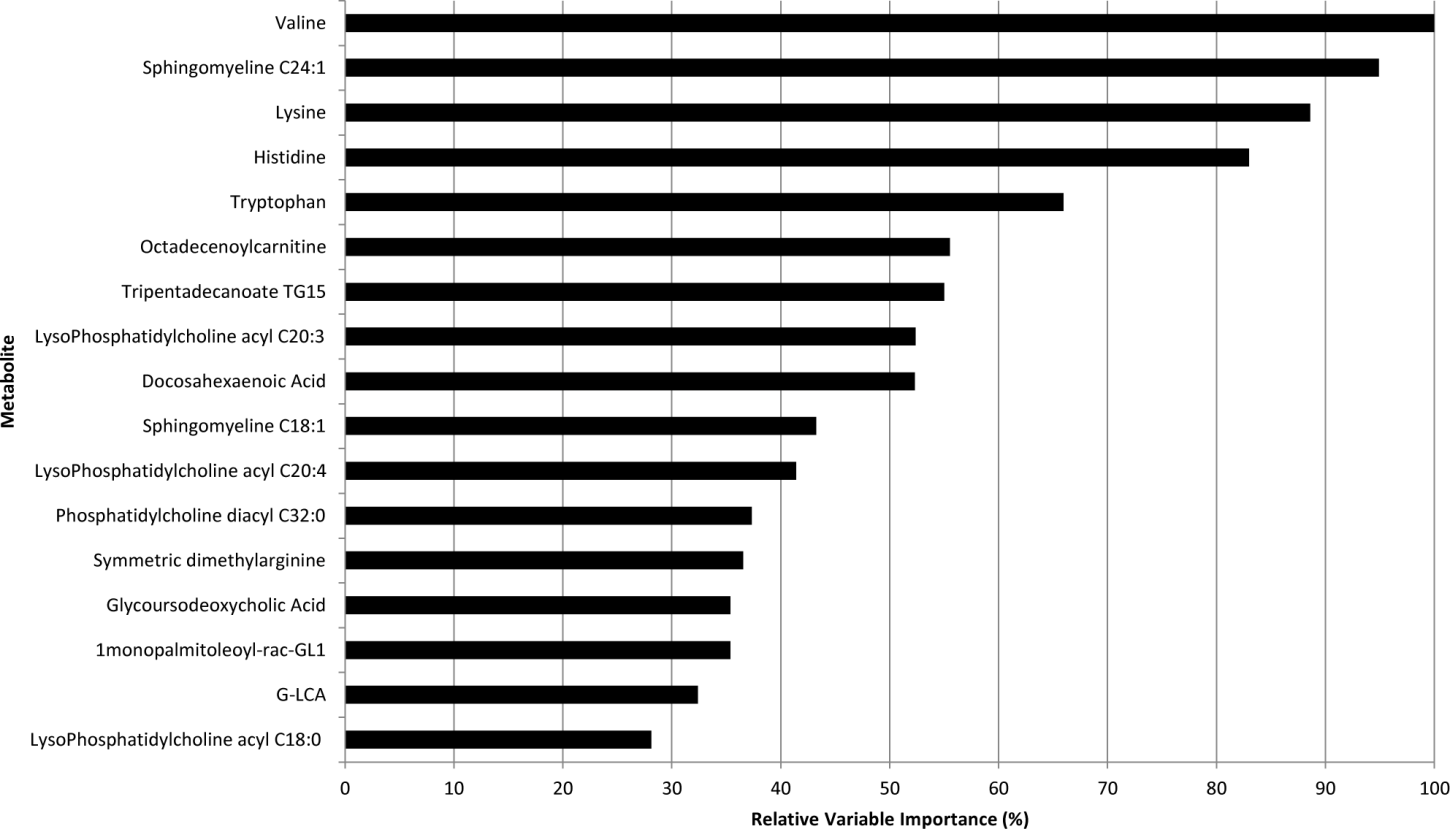
Relative importance values of the top variables (from the most to the less important) for the Random Forest (100,000 trees) which included metabolites only

Concentration value for each metabolite in PC patients were reported in Supplementary Table 1 whilst our metabolomic analysis was performed and described in the twin paper: Di Gangi I, Mazza T, Fontana A, Copetti M, Fusilli C, Ippolito A, Mattivi F, Latiano A, Andriulli A, Vrhovsek U and Pazienza V. “Metabolomic profile in pancreatic cancer patients: a consensus-based approach to identify highly discriminating metabolites”. Oncotarget 2016 IN PRESS. We found that Valine, Sphingomyeline C24:1, Lysine, Histidine, Tryptophan, Octadecenoylcarnitine, Tripentadecanoate TG15, LysoPhosphatidylcholine acyl C20:3, Docosahexaenoic Acid, Sphingomyeline C18:1, LysoPhosphatidylcholine acyl C20:4, Phosphatidylcholinediacyl C32:0, Symmetric dimethylarginine, Glycoursodeoxycholic Acid, 1monopalmitoleoyl-rac-GL1, G-LCA, LysoPhosphatidylcholine acyl C18:0 were the most important predictors of one year mortality risk (as shown in Figure 1 reporting from the most to the less important). Among metabolites detected in RSF, we found that Valine, Sphingomyeline C24:1, Lysine, Tripentadecanoate TG15 and Symmetric dimethylarginine were selected following the stepwise variable selection criterion (achieving a AIC of −5.04) and entered into the Weibull multivariable survival model. Univariable and multivariable two-order fractional polynomial models indicated a linear association between such metabolites and one-year mortality risk as more informative than any other non-linear association. Moreover, no significant improvement in model's goodness of fit was achieved when previous metabolites were considered into the RECPAM tree-structured analysis both as global and splitting variables.

Risk estimates for mortality at one-year were calculated, for each selected metabolite, by the multivariable Weibull model, along with empirical bootstrap 95%CI. Estimates were reported for each unitary increment in metabolites' SD: Valine (HR = 0.62, 95%CI: 0.11–1.71, for each SD of 98.57), Sphingomyeline C24:1 (HR = 2.66, 95%CI: 1.30–21.09, for each SD of 20.67), Lysine (HR = 0.36, 95%CI: 0.03–0.77, for each SD of 51.73), Tripentadecanoate TG15 (HR = 0.25, 95%CI: 0.01–0.82, for each SD of 2.88) and Symmetric dimethylarginine (HR = 2.24, 95%CI: 1.28–103.08, for each SD of 0.62).

For sake of completeness, we used patients' clinical information to investigate the major clinical predictor of mortality risk at one-year. Therefore, we run RSF analysis using clinical variables as candidates of each node split of each tree included into the forest. We found that lactate dehydrogenase, age, smoking habits and alcohol consumption resulted to best predict the one-year mortality risk (with a relative variable importance greater than 34%). Moreover, we found statistically significant positive correlations between the Lactate Dehydrogenase levels and the following metabolites: Octadecenoylcarnitine (*r* = 0.41, *p* = 0.044), Docosahexaenoic Acid (*r* = 0.45, *p* = 0.023), Symmetric dimethylarginine (*r* = 0.46, *p* = 0.022), and significant negative correlations with Valine (*r* = −0.41, *p* = 0.043) and tripentadecanoate TG15 (*r* = −0.43, *p* = 0.030) levels, among PC patients.

The effect of such metabolites (on one-year mortality risk) remained statistically significant even after adjusting for age, smoking habits, alcohol consumption, disease duration and surgical intervention (main confounders) either including them one by one or all together into the multivariable Weibull model. Such associations retained the statistical significance value, even after adjusting for “the most important” clinical variables (i.e. Lactate Dehydrogenase, Age, Smoking habits, Alcohol consumption).

### Development of MRS formula

Finally, we used the parameter estimates from the multivariable Weibull model as stabilized weights to build MRS. Specifically, we found that $\hat{\lambda }=1.7376$ and $\hat{\gamma }=1.0769$, represented the scale and the shape parameters estimates of the baseline survivor function, (from the empty Weibull model) respectively. For each metabolite, we subtracted its sample mean (we defined it as scaled metabolite). Specifically, the sample mean values (μmol/L) were: 249.84 for Valine (Val), 23.93 for Sphingomyeline C24:1 (SMC24), 200.53 for Lysine (Lys), 8.46 for Tripentadecanoate TG15 (TG15), 0.76 for Symmetric dimethylarginine (SDMA).

The estimated regression coefficients for scaled metabolites (around their sample means) were the following ones: −0.005 for Val, 0.047 for SMC24, −0.020 for Lys, −0.483 for TG15, 1.314 for SDMA.

Therefore, the final MRS formula is computed as follows:

$$MRS=1-0.1632^{\exp(-0.005\cdot \text{Val}+0.047\cdot \text{SMC}24-0.020\cdot \text{Lys}-0.483\cdot \text{TG}15+1.314\cdot \text{SDMA})}$$

MRS provided an estimate of the individual prediction probability to have a death occurrence within one year of follow-up, depending on the individual levels of Val, SMC24, Lys, TG15 and SDMA collected at baseline. MRS achieved a high discriminatory power (i.e. survival c-statistic of 0.855, 95%CI: 0.816–0.894). For sake of completeness, the prognostic ability of TNM staging (one of the most important factor in determining prognosis and treatment options) alone was further evaluated, computing the modified c-statistic for censored survival data, using predicted probabilities at one-year in a multivariable Weibull model. It resulted that TNM achieved the a survival c-statistic of 0.591 (95%CI: 0.467–0.714), which was significantly lower than the c-statistic achieved by MRS only, suggesting an evident superiority (*p* < 0.001) for MRS to assign higher mortality risk in patients who will die and lower mortality risk in patients who will survive.

### Survival analysis

RECPAM method was used to identify optimal cut-offs for MRS which defined patients' subgroups for MRS with different mortality risk. The algorithm stopped at three subgroups (RECPAM classes), which achieved the minimum AIC. The reference class (Class 3) was represented by the subgroup with the lowest mortality, and all the HRs were estimated with respect to such reference class. Patients with MRS ≤ 0.50 represented the reference class, whereas patients with MRS > 0.88 represented the class with the highest mortality risk (Class 1, HR = 30.24, 95% CI: 6.01–152.11, *p* < 0.001). Patients with 0.50 < MRS ≤ 0.88 represented the intermediate risk class (Class 2, HR = 4.44, 95% CI: 0.79–24.97, *p* = 0.091).

**Figure 2:**
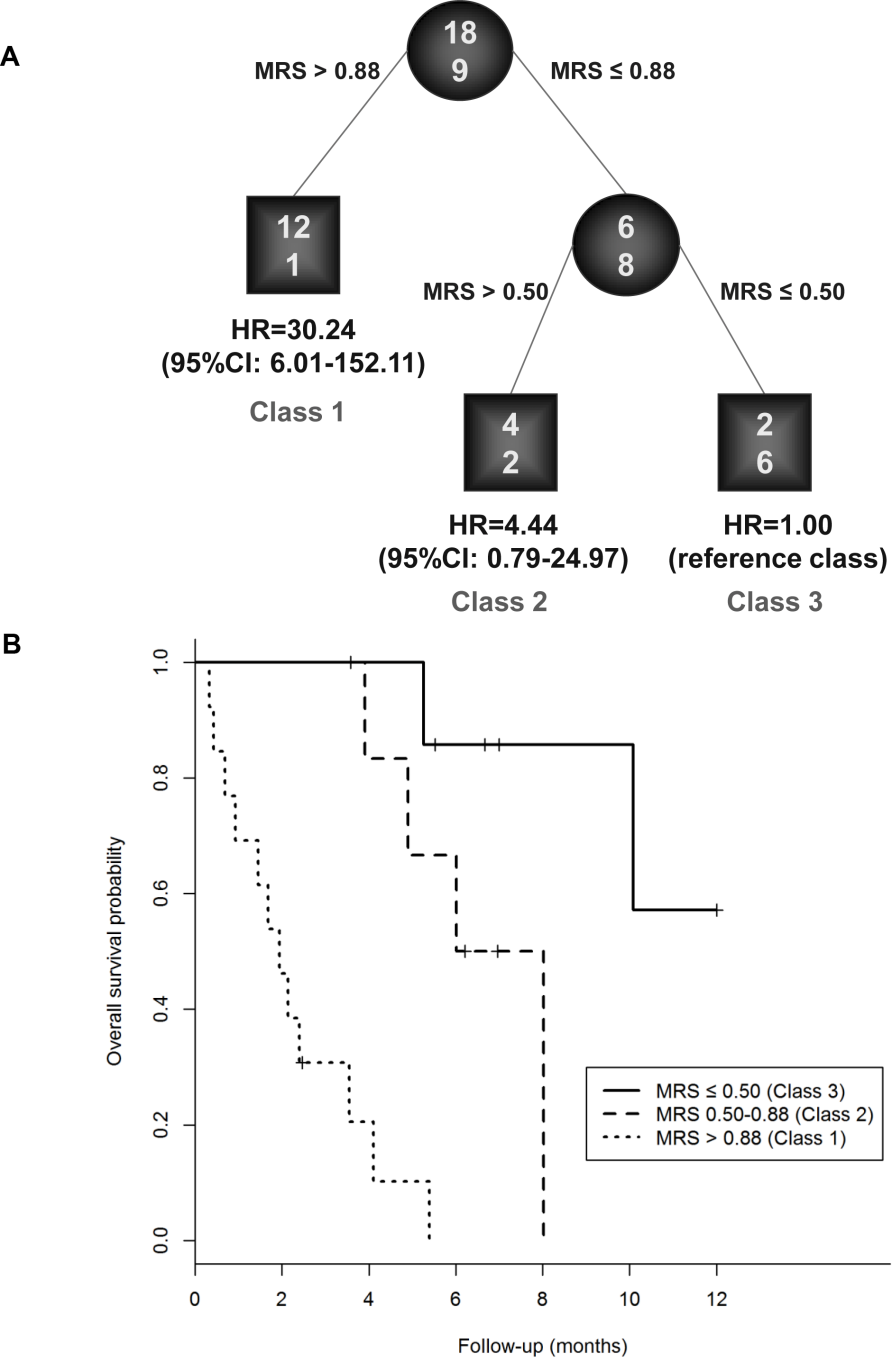
Identification of patients' subgroups at different risks for one-year mortality: RECPAM survival tree (A) and Kaplan-Meier curves (B) for patients who belonged to low, intermediate and high risk classes for MRS RECPAM analysis identified patient subgroups at different risks for mortality. The tree-growing algorithm modeled hazard ratios after a Weibull model. MRS was used as splitting variable and splits are shown between branches, while condition sending patients to left or right sibling is on relative branch. Class 3 with lowest mortality was reference category (HR = 1). Circles indicate subgroups of patients. Squares indicate patient subgroup RECPAM class. Numbers inside circles and squares represent the number of events (top) and the number of non-events (bottom), respectively.

**Figure 3:**
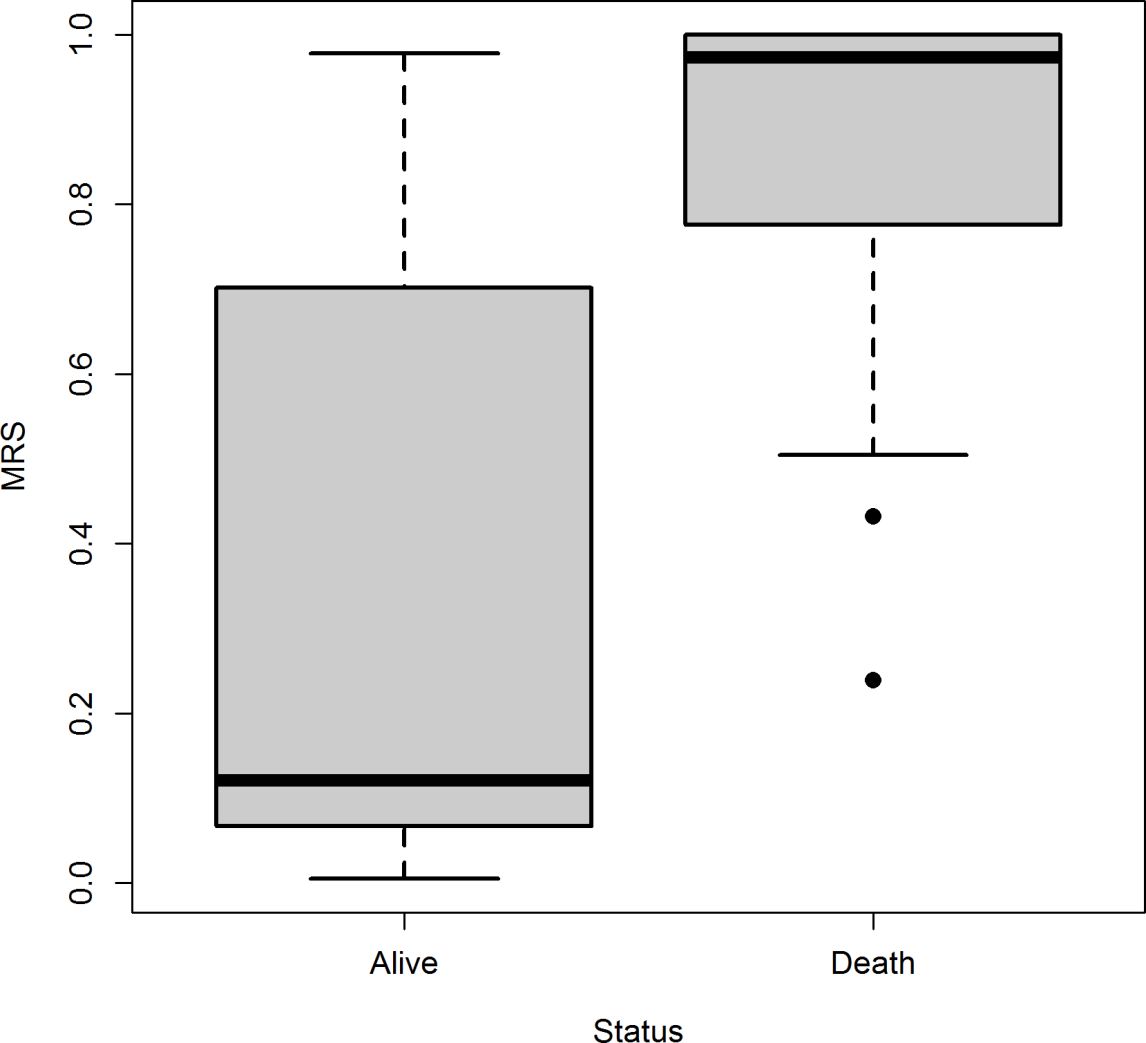
Boxplot of MRS for patients who survived and those who did not survive, separately

RECPAM tree along with Kaplan Maier survival curves for each RECPAM class are further reported in Figure 2 (A and B). MRS distributions are represented into a boxplot (Figure 3) for patients who survived and those who did not survive at the end of follow-up time.

## DISCUSSION

Recent studies performed in gemcitabine sensitive and in chemoresistant pancreatic cell lines by Fujimura and colleagues [19], observed significant differences in metabolites related to several metabolic pathways such as amino acids, nucleotides, energy, cofactors, and vitamin pathways. These findings were supportive of the experimental hypothesis of characteristic features in the metabolome of gemcitabine sensitive and resistant cells and corroborated the understanding of resistance mechanisms in pancreatic cancer. For instance, the altered concentration of arginine reported by Bathe [20] may be crucial for the increase of eNOS levels and NO production which in turn triggers hCHOP-C/EBPa complex formation and reduces hENT1 expression [21]. The latter plays an important role in predicting clinical outcomes in PDAC [17]. Decreased hENT1 levels may also be triggered to a high glucose concentration [22, 23], which were found to be altered in pancreatic cancer patients' urine and serum by Napoli et al. [24], Davis et al. [25], and Bathe et al. [20]. Creatine, creatinine, aspartate, and citrulline are representative metabolites related to urea cycle and are involved in the production of nitric oxide (NO). This pathway is related to glutamate and proline metabolisms, regulating bioenergetics and redox status [26]. Most cancer cells demonstrate an increase in glucose uptake, a higher rate of glycolysis, and an increase in lactate secretion despite the presence of oxygen, a phenomenon known as the Warburg effect [27].

Here we report the analysis of potential changes in the concentration of metabolites in serum samples of pancreatic cancer patients in order to find possible associations between the mutual concentration of these metabolites and clinical pathological features in a selected and well characterized cohort of patients with PDAC. Moreover, among patients with pancreatic adenocarcinoma, we identified all those metabolites which might be potentially predictive of patient's one-year mortality, with the building of a Metabolites Risk Score (MRS). Our method consisted in three steps: preprocessing, selection and classification and it can be adapted to other metabolomics/proteomics/genomics data analysis studies. Furthermore, metabolites selected in this process may shed lights on the pathways and mechanisms underpinning pancreatic cancer onset and/or development. The major limitation of this study was the lack of an external validation cohort of PDAC patients, which would let to corroborate or disprove the performance of MRS we achieved using the development cohort of 27 patients. In conclusion the implementation of our MRS formula may help prioritize the use of available resources for targeting aggressive preventive and treatment strategies in a subset of very high-risk individuals.

## MATERIALS AND METHODS

### Study population and inclusion criteria

Forty patients with pancreatic adenocarcinoma were consecutively recruited at the Scientific Institute “Casa Sollievo della Sofferenza” Hospital in San Giovanni Rotondo (Apulia, Italy) from May to November 2012 for a serum blood sample collection. All patients signed an informed consent approved by the local Ethics Committee. The consent included collection of serum sample and clinical patient's features. After excluding patients *a)* who had missing data for one or more baseline clinical features (*n* = 10) and *b)* whose information on vital status at follow-up was not available (*n* = 3), 27 patients with pancreatic adenocarcinoma (67.5% of the initial cohort) constituted the eligible sample for the present analysis. PC patients' clinical characteristics evaluated at baseline were reported in Table 1.

**Table 1:** Baseline clinical-pathological characteristics of all 27 patients with pancreatic cancer

**Age at diagnosis** (yrs), *median (Q1–Q3)*	66 (56–76)
**Gender,** *n males (%)*	13 (48.15)
**BMI** (kg/m^2^), *median (Q1*–*Q3)*	24.8 (22.96–29.23)
**Waist** (cm), *median (Q1*–*Q3)*	101 (88–109)
**Presence of diabete,** *n (%)*	10 (37.04)
**Presence of hypertension, *n (%)***	9 (33.33)
**Glycemia** (mg/dl), *median (Q1*–*Q3)*	123 (97–145)
**Triglycerides** (mg/dl), *median (Q1*–*Q3)*	119.5 (83.5–304)
**Total cholesterol** (mg/dl), *median (Q1*–*Q3)*	150.5 (127.5–215.5)
**Lactate dehydrogenase** (UI/l), *median (Q1*–*Q3)*	348 (260–372)
**Smoking habits,** *n (%*)	
Yes	6 (22.22)
No	14 (51.85)
Ex	7 (25.93)
**Alcohol consumption**, *n (%)*	
No drinker	8 (30.77)
Occasional drinker	10 (38.46)
Moderate drinker	5 (19.23)
Heavy drinker	3 (11.54)
Missing information	1
**Coffee consumption,** *n (%)*	
No	5 (20)
Yes	20 (80)
Missing information	2
**Nr. drinked cups of coffee/day,** *median (Q1*–*Q3)*	2 (1–4)
**Presence of (any) familiarity,** *n (%)*	11 (40.74)
**CA 19-9** (U/ml), *median (Q1*–*Q3)*	1839.5 (860–3398)
**Jaundice,** *n (%)*	
No	9 (39.13)
Yes	14 (60.87)
Missing information	4
**Pain,** *n (%)*	
No	7 (28)
Yes	18 (72)
Missing information	2
**Weight loss,** *n (%)*	
No	3 (18.75)
Yes	13 (81.25)
Missing information	11
**Surgical resection,** *n (%)*	7 (25.93)
**Tumour localization,** *n (%)*	
Body/tail	5 (18.52)
Head	22 (81.48)
**T: Tumour size,** *n (%)*	
Tx	4
T1	1 (4.35)
T2	2 (8.7)
T3	11 (47.83)
T4	9 (39.13)
**N: regional lymph nodes,** *n (%)*	
Nx	9
N0	4 (22.22)
N1	13 (72.22)
N2	1 (5.56)
**M: distant metastasis,** *n (%)*	
Mx	7
M0	10 (50)
M1	10 (50)
**Margins of resection,** *n (%)*	
Rx	1
R0	4 (15.38)
R1	2 (7.69)
R2	20 (76.92)
**Presence of vascular invasion,** *n (%)*	6 (22.22)
**Presence of chemotherapy**, *n (%)*	13 (48.15)
**Disease duration** (yrs)*, *median (Q1*–*Q3)*	0.45 (0.1–1.11)
**Time from diagnosis** (yrs)**, *median (Q1*–*Q3)*	0.1 (0.06–0.45)
**Overall follow-up time** (yrs), *median (Q1*–*Q3)*	0.34 (0.16–0.56)
**Mortality rate overall,** *ev/py (ir%)^*	18/45 (39.7)
**Mortality rate at one-year,** *ev/py (ir%)^*	18/41 (43.5)

* Time from symptoms to blood collection;

** Time from diagnosis to blood collection;

^ events/person-years, incidence rate per 100 person-years; Q1–Q3: first-third quartiles.

### Random survival forest analysis

This appealing method is widely used to identify, among all available covariates (i.e., all metabolites), the ones which were most associated to a specific dependent variable of interest (i.e. one-year mortality risk). This method is also known to provide extremely robust results, especially in context of high dimensional dataset. To our purpose, we built 100,000 survival trees. The training set used to grow each tree is a 0.632+ bootstrap resample of the observations [28]. Trees were allowed to grow to their full size without pruning. The best split at each node of the tree included into the forest was selected from a random subset of covariates. At each node, the split which maximizes the log-rank score is chosen. The left-out (i.e. “out of bag”) observations were then used to obtain the classification error of the considered tree. The Random Survival Forest (RFS) goodness of fit was assessed averaging the individual tree classification errors. One of the most relevant strength of RSF analysis is its capability to impute missing data. Prior to splitting a node, missing data for a variable is imputed by randomly drawing values from non-missing in-bag data. Imputed data is however not used to calculate the split-statistic which uses non-missing data only. Following a node split, imputed data are reset to missing and the process is repeated until terminal nodes are reached. Missing data in terminal nodes are imputed using OOB non-missing terminal node data. Furthermore, the RSF framework estimates the importance of each covariate achieved to discriminate the dependent variable by looking at how much the classification error increases when out of bag data for that variable are permuted, while all others are left unchanged. The variables' importance is ranked by assigning to each covariate a score based on the ability to predict correctly the dependent variable according to the increase of classification error when values of that covariate in a node were randomly permuted. Furthermore, the relative variables' importance is defined, for each covariate, as the ratio of the score assigned to the specific covariate and the score assigned to the first ranked covariate (which, by definition, is the most important one). The Breiman-Cutler measure is used. This measure is constructed by permuting the values of each variable of the test set, recording the prediction and comparing it with the un-permuted test set prediction of the variable. Therefore, it is the increase in the percentage of times a test set is misclassified when the variable is permuted.

### Statistical analysis

Clinical patients' characteristics and metabolites concentration levels were reported as medians (along with lower-upper quartiles) or frequencies and percentages for continuous and categorical variables, respectively. The overall mortality rate was calculated as the number of death events per 100 person-years.

Association between clinical variables and metabolites concentration levels were assessed using Mann-Whitney *U* test (or Kruskal-Wallis when appropriate) or Spearman correlation coefficient for categorical and continuous clinical variables, respectively.

The overall survival was defined as the time between baseline and event (i.e. death) dates; for subjects who did not experience the event, survival time was censored at the time of the last available follow-up information.

Disease duration was defined as the time between the date of diagnosis (which precedes the baseline) and the baseline date (i.e. the date of serum blood collection).

Time-to-death analyses were performed using Weibull survival regression models, and risks were reported as hazard ratios (HR) along with their 95% confidence intervals (95% CI). For metabolite concentrations, HR were reported for each unitary increase of their standard deviation (SD). The appropriateness of the Weibull parameterization assumption for the baseline hazard was graphically ascertained, plotting the natural logarithm of the Kaplan-Meier survival estimate (which their values were reverted and logarithm transformed as well) against the logarithm of follow-up time. To build a Metabolites Risk Score (MRS) which best predicted patient's one-year mortality risk and comprised a combination of the highest associated metabolites (i.e. predictors), we followed a three-step process:

*Step 1* – A Random Survival Forest (RSF) analysis [29] [*see Random Survival Forest analysis section*] was performed using metabolites concentration levels as continuous splitting variables for the building of the forest. All missing values were internally imputed by RSF for all covariates as long as their missing rate was lower than 25%. When the missing rate is higher than 25%, covariates were excluded from any further statistical analysis [30].

*Step 2* – From RSF variable importance, we selected “the most important” metabolites which achieved at least 20% of relative variable importance. To estimate the proper functional relationship between such predictors and one-year mortality risk, all the selected metabolites were included as covariates into a Weibull survival regression and, following a stepwise variable selection criterion, we identified the strongest predictors which were linearly associated to one-year mortality risk (significance level for entry into the model: *p* = 0.10, significance level for staying into the model: *p* = 0.05). The overall models' goodness of fit was evaluated by the Akaike's information criterion (AIC). At each iteration step of the stepwise procedure, all predictors which minimize AIC were consecutively included into the model, after 10,000 bootstrap resamplings. To provide more robustness of the association between the effect of stepwise-selected metabolites and one-year mortality risk, we further evaluated possible non-linear relationship, including metabolites as non-linear trend components into two-order fractional polynomial models [31]. Furthermore, we checked possible interactions between selected metabolites using RECursive Partitioning and AMalgamation (RECPAM) method [17, 32].

*Step 3* – We finally used the regression coefficients estimates from the final multivariable Weibull survival model as weights to build the MRS. The “stability” of these weights was assessed performing 10,000 bootstrap resamplings where, at each iteration step, a multivariable Weibull model was run on the bootstrapped sample and the estimates of regression coefficients were stored. Thereafter, the empirical distribution of each weight was determined along with the corresponding distribution-based 95%CI. Therefore, assuming that the survival time is a random variable which follows a Weibull distribution with scale and shape parameters of *λ* and *γ* respectively, the patient's baseline survivor function *S*_0_ (*t*) is characterized by the following formula: *S*_0_ (*t*) = exp {−(*λt*)^*γ*^}, where t is any non-negative specific survival time point. Following the assumption of proportional hazards (i.e., the effect of the covariates increase or decrease the hazard by a proportionate amount at all durations) and having considered we want to make a mortality risk prediction at one-year (i.e., *t* = 1), we built the MRS as follows:

$$\text{MRS}=1-S_{0}{\left(t\right)}^{e^{\sum_{i=1}^{p}{\hat{\beta }}_{i}(X_{i}-{\bar{X}}_{i})}}=1-\text{exp}{\left\{-{\hat{\lambda }}^{\hat{\gamma }}\right\}}^{{}^{e^{\sum_{i=1}^{p}{\hat{\beta }}_{i}(X_{i}-{\bar{X}}_{i})}}}$$

Where $\hat{\lambda }=1/\exp(\hat{\beta_{0}})$ and $\hat{\gamma }$ are the Weibull scale and shape parameters estimated from the empty Weibull model (without covariates), respectively, and $\hat{\beta_{0}}$ the model's intercept and $\hat{\beta_{i}}$ is the i-th regression coefficient for the i-th *X_i_* metabolite (estimated from the multivariable Weibull model), which values were centered around their sample mean. The obtained MRS ranged from 0 (lowest mortality risk within one year of follow-up) to 1 (highest mortality risk within one year of follow-up). Furthermore, RECPAM method was used to identify optimal cut-offs for MRS which defined patients' subgroups at different mortality risk.

Models' discrimination, i.e. the ability to distinguish subjects who will develop an event from those who will not, was assessed by computing the modified C-statistic for censored survival data [33], using predicted probabilities at one-year.

All statistical analyses were performed using SAS Release 9.3 (SAS Institute, Cary, NC, USA) and R software (ver. 2.15, packages: ‘randomforestSRC’, ‘survival’, ‘MASS’, ‘mpf’, ‘boot’).

## SUPPLEMENTARY MATERIALS TABLE



## References

[1] Jemal A, Siegel R, Ward E, Hao Y, Xu J, Thun MJ (2009). Cancer statistics, 2009. CA Cancer J Clin.

[2] Urayama S, Zou W, Brooks K, Tolstikov V (2010). Comprehensive mass spectrometry based metabolic profiling of blood plasma reveals potent discriminatory classifiers of pancreatic cancer. Rapid Commun Mass Spectrom.

[3] Ranganathan P, Harsha HC, Pandey A (2009). Molecular alterations in exocrine neoplasms of the pancreas. Arch Pathol Lab Med.

[4] Pazienza V, Tavano F, Benegiamo G, Vinciguerra M, Burbaci FP, Copetti M, di Mola FF, Andriulli A, di Sebastiano P (2012). Correlations among PPARgamma, DNMT1, and DNMT3B Expression Levels and Pancreatic Cancer. PPAR Res.

[5] Jordheim LP, Seve P, Tredan O, Dumontet C (2011). The ribonucleotide reductase large subunit (RRM1) as a predictive factor in patients with cancer. Lancet Oncol.

[6] Akita H, Zheng Z, Takeda Y, Kim C, Kittaka N, Kobayashi S, Marubashi S, Takemasa I, Nagano H, Dono K, Nakamori S, Monden M, Mori M (2009). Significance of RRM1 and ERCC1 expression in resectable pancreatic adenocarcinoma. Oncogene.

[7] Fujita H, Ohuchida K, Mizumoto K, Itaba S, Ito T, Nakata K, Yu J, Kayashima T, Souzaki R, Tajiri T, Manabe T, Ohtsuka T, Tanaka M (2010). Gene expression levels as predictive markers of outcome in pancreatic cancer after gemcitabine-based adjuvant chemotherapy. Neoplasia.

[8] Nakahira S, Nakamori S, Tsujie M, Takahashi Y, Okami J, Yoshioka S, Yamasaki M, Marubashi S, Takemasa I, Miyamoto A, Takeda Y, Nagano H, Dono K (2007). Involvement of ribonucleotide reductase M1 subunit overexpression in gemcitabine resistance of human pancreatic cancer. Int J Cancer.

[9] Xie H, Jiang W, Jiang J, Wang Y, Kim R, Liu X, Liu X (2013). Predictive and prognostic roles of ribonucleotide reductase M1 in resectable pancreatic adenocarcinoma. Cancer.

[10] Ohhashi S, Ohuchida K, Mizumoto K, Fujita H, Egami T, Yu J, Toma H, Sadatomi S, Nagai E, Tanaka M (2008). Down-regulation of deoxycytidine kinase enhances acquired resistance to gemcitabine in pancreatic cancer. Anticancer Res.

[11] Perez-Torras S, Garcia-Manteiga J, Mercade E, Casado FJ, Carbo N, Pastor-Anglada M, Mazo A (2008). Adenoviral-mediated overexpression of human equilibrative nucleoside transporter 1 (hENT1) enhances gemcitabine response in human pancreatic cancer. Biochem Pharmacol.

[12] Saiki Y, Yoshino Y, Fujimura H, Manabe T, Kudo Y, Shimada M, Mano N, Nakano T, Lee Y, Shimizu S, Oba S, Fujiwara S, Shimizu H (2012). DCK is frequently inactivated in acquired gemcitabine-resistant human cancer cells. Biochem Biophys Res Commun.

[13] Giovannetti E, Del Tacca M, Mey V, Funel N, Nannizzi S, Ricci S, Orlandini C, Boggi U, Campani D, Del Chiaro M, Iannopollo M, Bevilacqua G, Mosca F (2006). Transcription analysis of human equilibrative nucleoside transporter-1 predicts survival in pancreas cancer patients treated with gemcitabine. Cancer Res.

[14] Greenhalf W, Ghaneh P, Neoptolemos JP, Palmer DH, Cox TF, Lamb RF, Garner E, Campbell F, Mackey JR, Costello E, Moore MJ, Valle JW, McDonald AC (2014). Pancreatic cancer hENT1 expression and survival from gemcitabine in patients from the ESPAC-3 trial. J Natl Cancer Inst.

[15] Marechal R, Bachet JB, Mackey JR, Dalban C, Demetter P, Graham K, Couvelard A, Svrcek M, Bardier-Dupas A, Hammel P, Sauvanet A, Louvet C, Paye F (2012). Levels of gemcitabine transport and metabolism proteins predict survival times of patients treated with gemcitabine for pancreatic adenocarcinoma. Gastroenterology.

[16] McAllister F, Pineda DM, Jimbo M, Lal S, Burkhart RA, Moughan J, Winter KA, Abdelmohsen K, Gorospe M, Acosta Ade J, Lankapalli RH, Winter JM, Yeo CJ (2014). dCK expression correlates with 5-fluorouracil efficacy and HuR cytoplasmic expression in pancreatic cancer: a dual-institutional follow-up with the RTOG 9704 trial. Cancer Biol Ther.

[17] Tavano F, Fontana A, Pellegrini F, Burbaci FP, Rappa F, Cappello F, Copetti M, Maiello E, Lombardi L, Graziano P, Vinciguerra M, di Mola FF, di Sebastiano P (2014). Modeling interactions between Human Equilibrative Nucleoside Transporter-1 and other factors involved in the response to gemcitabine treatment to predict clinical outcomes in pancreatic ductal adenocarcinoma patients. J Transl Med.

[18] Mohelnikova-Duchonova B, Melichar B (2013). Human equilibrative nucleoside transporter 1 (hENT1): do we really have a new predictive biomarker of chemotherapy outcome in pancreatic cancer patients?. Pancreatology.

[19] Fujimura Y, Ikenaga N, Ohuchida K, Setoyama D, Irie M, Miura D, Wariishi H, Murata M, Mizumoto K, Hashizume M, Tanaka M (2014). Mass spectrometry-based metabolic profiling of gemcitabine-sensitive and gemcitabine-resistant pancreatic cancer cells. Pancreas.

[20] Bathe OF, Shaykhutdinov R, Kopciuk K, Weljie AM, McKay A, Sutherland FR, Dixon E, Dunse N, Sotiropoulos D, Vogel HJ (2011). Feasibility of identifying pancreatic cancer based on serum metabolomics. Cancer Epidemiol Biomarkers Prev.

[21] Pandolfi A, Di Pietro N (2010). High glucose, nitric oxide, and adenosine: a vicious circle in chronic hyperglycaemia?. Cardiovasc Res.

[22] D'Aronzo M, Vinciguerra M, Mazza T, Panebianco C, Saracino C, Pereira SP, Graziano P, Pazienza V (2015). Fasting cycles potentiate the efficacy of gemcitabine treatment in *in vitro* and *in vivo* pancreatic cancer models. Oncotarget.

[23] Farrell JJ, Elsaleh H, Garcia M, Lai R, Ammar A, Regine WF, brams R, Benson AB, Macdonald J, Cass CE, Dicker AP, Mackey JR (2009). Human equilibrative nucleoside transporter 1 levels predict response to gemcitabine in patients with pancreatic cancer. Gastroenterology.

[24] Napoli C, Sperandio N, Lawlor RT, Scarpa A, Molinari H, Assfalg M (2012). Urine metabolic signature of pancreatic ductal adenocarcinoma by (1) h nuclear magnetic resonance: identification, mapping, and evolution. J Proteome Res.

[25] Davis VW, Schiller DE, Eurich D, Bathe OF, Sawyer MB (2012). Pancreatic ductal adenocarcinoma is associated with a distinct urinary metabolomic signature. Ann Surg Oncol.

[26] Rath M, Muller I, Kropf P, Closs EI, Munder M (2014). Metabolism via Arginase or Nitric Oxide Synthase: Two Competing Arginine Pathways in Macrophages. Front Immunol.

[27] Hsu PP, Sabatini DM (2008). Cancer cell metabolism: Warburg and beyond. Cell.

[28] Efron B, Tibshirani RJ (1997). Improvements on cross-validation: the 632+ bootstrap method. Journal of the American Statistical Association.

[29] Breiman L (2001). Random Forests. Machine Learning.

[30] Barzi F, Woodward M (2004). Imputations of missing values in practice: results from imputations of serum cholesterol in 28 cohort studies. Am J Epidemiol.

[31] Royston P, Altman D (1994). Regression using fractional polynomials of continuous covariates. Journal of the Royal Statistical Society Series C (Applied Statistics).

[32] Ciampi A (1991). Generalized Regression Trees. Computational Satistics and Data Analysis.

[33] Pencina MJ, D'Agostino RB (2004). Overall C as a measure of discrimination in survival analysis: model specific population value and confidence interval estimation. Stat Med.

