# Supplementary material for: Development of a metabolites risk score for one-year mortality risk prediction in pancreatic adenocarcinoma patients
Source: Oncotarget. 2016 Feb 1;7(8):8968–78. doi: 10.18632/oncotarget.7108 (PMC4891018; doi:10.18632/oncotarget.7108)
Supplement: Supplementary file 1 [file oncotarget-07-8968-s001.pdf]

Development of a metabolites risk score for one–year mortality risk prediction in pancreatic adenocarcinoma patients

Supplementary Materials

Supplementalry Table 1: Concentration levels (µmol/L) for each metabolite of patients with pancreatic cancer (median, first–third quartiles)

Metabolite	*N* of available patients	
C0	*N* = 27	39.93 (32.76–46.33)
C10:2	*N* = 27	0.05 (0.04–0.09)
C12–DC	*N* = 27	0.06 (0.03–0.08)
C14:1	*N* = 27	0.28 (0.22–0.34)
C14:2	*N* = 27	0.05 (0.03–0.06)
C14:2–OH	*N* = 27	0.02 (0.01–0.02)
C16	*N* = 27	0.10 (0.06–0.16)
C16–OH	*N* = 27	0.02 (0.01–0.03)
C16:1	*N* = 27	0.05 (0.04–0.06)
C16:2	*N* = 27	0.02 (0.01–0.03)
C16:2–OH	*N* = 27	0.02 (0.02–0.03)
C18	*N* = 27	0.05 (0.02–0.08)
C18:1	*N* = 27	0.25 (0.17–0.30)
C18:1–OH	*N* = 27	0.03 (0.02–0.04)
C18:2	*N* = 27	0.07 (0.04–0.09)
C2	*N* = 27	6.66 (4.14–9.42)
C3	*N* = 16	0.27 (0.18–0.42)
C3–DC (C4–OH)	*N* = 27	0.13 (0.09–0.17)
C3–OH	*N* = 27	0.04 (0.03–0.05)
C3:1	*N* = 27	0.02 (0.01–0.02)
C4:1	*N* = 27	0.02 (0.02–0.03)
C5	*N* = 27	0.12 (0.09–0.18)
C5–OH (C3–DC–M)	*N* = 27	0.10 (0.08–0.13)
C5:1	*N* = 27	0.02 (0.02–0.03)
C5:1–DC	*N* = 27	0.03 (0.02–0.04)
C6:1	*N* = 27	0.05 (0.04–0.06)
C7–DC	*N* = 27	0.03 (0.02–0.04)
C9	*N* = 27	0.04 (0.03–0.06)
Ala	*N* = 27	501.70 (390.60–759.80)
Arg	*N* = 27	120.90 (103.40–151.80)
Asn	*N* = 27	62.50 (55.10–67.30)
Gln	*N* = 27	259.10 (194.50–367.80)
Glu	*N* = 27	110.50 (77.60–176.20)

Gly	*N* = 27	263.30 (234.10–303.90)
His	*N* = 27	83.20 (63.30–94.80)
Ile	*N* = 27	88.20 (70.40–109.40)
Leu	*N* = 27	23.70 (1.50–68.80)
Lys	*N* = 27	200.70 (163.90–251.10)
Met	*N* = 27	32.80 (28.30–39.70)
Orn	*N* = 27	92.20 (70.00–117.30)
Phe	*N* = 27	108.10 (88.50–127.60)
Pro	*N* = 27	197.60 (161.00–275.20)
Ser	*N* = 26	129.90 (92.00–172.00)
Thr	*N* = 27	211.90 (142.10–310.20)
Trp	*N* = 27	57.30 (35.90–64.60)
Tyr	*N* = 27	69.00 (64.10–86.80)
Val	*N* = 27	217.40 (189.70–295.60)
alpha–AAA	*N* = 27	8.90 (0.30–9.00)
Creatinine	*N* = 27	172.30 (123.40–225.40)
Kynurenine	*N* = 27	1.90 (0.90–2.80)
Putrescine	*N* = 27	0.30 (0.03–0.40)
SDMA	*N* = 27	0.60 (0.40–0.90)
Serotonin	*N* = 27	0.60 (0.20–1.20)
Spermidine	*N* = 27	0.30 (0.30–0.50)
t4–OH–Pro	*N* = 27	11.10 (6.90–19.00)
Taurine	*N* = 27	184.90 (120.50–211.90)
lysoPC a C16:0	*N* = 27	82.71 (44.88–118.54)
lysoPC a C16:1	*N* = 27	2.69 (1.98–3.74)
lysoPC a C17:0	*N* = 27	1.43 (0.94–2.21)
lysoPC a C18:0	*N* = 27	18.25 (10.47–30.39)
lysoPC a C18:1	*N* = 27	21.32 (14.86–26.36)
lysoPC a C18:2	*N* = 27	15.47 (10.26–23.91)
lysoPC a C20:3	*N* = 27	2.25 (1.57–2.83)
lysoPC a C20:4	*N* = 27	5.75 (4.26–8.68)
lysoPC a C24:0	*N* = 27	0.71 (0.53–0.98)
lysoPC a C26:0	*N* = 27	1.33 (1.13–1.59)
lysoPC a C26:1	*N* = 27	1.03 (0.72–1.30)
lysoPC a C28:0	*N* = 27	1.57 (1.06–1.74)
lysoPC a C28:1	*N* = 27	1.77 (1.27–2.32)
PC aa C24:0	*N* = 27	0.28 (0.22–0.36)
PC aa C26:0	*N* = 27	1.56 (1.37–2.25)
PC aa C28:1	*N* = 27	1.30 (0.98–1.54)
PC aa C30:0	*N* = 27	2.97 (1.96–4.89)
PC aa C32:0	*N* = 27	9.49 (7.87–22.10)
PC aa C32:1	*N* = 27	11.62 (7.53–25.94)
PC aa C32:3	*N* = 27	0.17 (0.13–0.27)
PC aa C34:1	*N* = 27	191.71 (149.76–371.64)

PC aa C34:2	$N = 27$	205.54 (170.16–312.52)
PC aa C34:3	$N = 27$	5.93 (4.38–10.94)
PC aa C34:4	$N = 27$	0.53 (0.33–0.87)
PC aa C36:0	$N = 27$	4.19 (3.01–4.72)
PC aa C36:1	$N = 27$	30.17 (22.97–45.49)
PC aa C36:2	$N = 27$	107.98 (83.72–155.78)
PC aa C36:3	$N = 27$	69.72 (51.17–94.08)
PC aa C36:4	$N = 27$	112.84 (90.09–209.62)
PC aa C36:5	$N = 27$	6.10 (4.18–9.08)
PC aa C36:6	$N = 27$	0.33 (0.28–0.55)
PC aa C38:0	$N = 27$	1.81 (1.28–2.44)
PC aa C38:3	$N = 27$	23.44 (16.52–37.79)
PC aa C38:4	$N = 27$	54.80 (38.32–73.86)
PC aa C38:5	$N = 27$	22.97 (16.10–31.54)
PC aa C38:6	$N = 27$	35.19 (25.52–60.44)
PC aa C40:1	$N = 27$	0.45 (0.40–0.50)
PC aa C40:2	$N = 27$	0.47 (0.34–0.64)
PC aa C40:3	$N = 27$	0.49 (0.35–0.66)
PC aa C40:4	$N = 27$	1.82 (1.48–2.92)
PC aa C40:5	$N = 27$	4.91 (3.57–7.65)
PC aa C40:6	$N = 27$	10.42 (8.62–17.95)
PC aa C42:0	$N = 27$	0.38 (0.30–0.45)
PC aa C42:1	$N = 27$	0.20 (0.16–0.25)
PC aa C42:2	$N = 27$	0.17 (0.14–0.25)
PC aa C42:4	$N = 27$	0.20 (0.15–0.26)
PC aa C42:5	$N = 27$	0.22 (0.18–0.36)
PC aa C42:6	$N = 27$	0.30 (0.23–0.44)
PC ae C30:0	$N = 27$	0.41 (0.36–0.59)
PC ae C30:1	$N = 27$	0.38 (0.32–0.49)
PC ae C30:2	$N = 27$	0.23 (0.18–0.29)
PC ae C32:1	$N = 27$	1.55 (1.30–2.53)
PC ae C32:2	$N = 27$	0.39 (0.31–0.49)
PC ae C34:0	$N = 27$	1.06 (0.75–1.73)
PC ae C34:1	$N = 27$	6.49 (4.91–11.92)
PC ae C34:2	$N = 27$	4.60 (3.19–6.35)
PC ae C34:3	$N = 27$	2.33 (1.70–3.37)
PC ae C36:0	$N = 27$	1.29 (0.94–2.16)
PC ae C36:1	$N = 27$	8.43 (6.16–13.24)
PC ae C36:2	$N = 27$	6.31 (5.21–9.44)
PC ae C36:3	$N = 27$	3.17 (2.08–4.19)
PC ae C36:4	$N = 27$	7.39 (5.80–10.28)
PC ae C36:5	$N = 27$	6.02 (4.49–7.23)
PC ae C38:0	$N = 27$	0.93 (0.64–1.46)
PC ae C38:1	$N = 27$	2.54 (1.89–3.45)

PC ae C38:2	*N* = 27	2.17 (1.71–3.09)
PC ae C38:3	*N* = 27	3.70 (2.63–4.86)
PC ae C38:4	*N* = 27	6.75 (5.65–10.06)
PC ae C38:5	*N* = 27	9.61 (8.00–12.26)
PC ae C38:6	*N* = 27	3.10 (2.71–3.99)
PC ae C40:1	*N* = 27	0.86 (0.61–1.07)
PC ae C40:2	*N* = 27	1.32 (0.94–1.69)
PC ae C40:3	*N* = 27	1.17 (0.92–1.50)
PC ae C40:4	*N* = 27	1.66 (1.20–2.03)
PC ae C40:5	*N* = 27	2.47 (1.94–3.21)
PC ae C40:6	*N* = 27	2.42 (1.84–2.88)
PC ae C42:1	*N* = 27	0.28 (0.23–0.43)
PC ae C42:2	*N* = 27	0.35 (0.26–0.42)
PC ae C42:3	*N* = 27	0.43 (0.34–0.54)
PC ae C42:4	*N* = 27	0.52 (0.42–0.68)
PC ae C42:5	*N* = 27	1.39 (1.12–1.81)
PC ae C44:3	*N* = 27	0.11 (0.09–0.15)
PC ae C44:4	*N* = 27	0.25 (0.20–0.33)
PC ae C44:5	*N* = 27	1.31 (0.99–1.58)
PC ae C44:6	*N* = 27	0.70 (0.53–0.92)
SM (OH) C14:1	*N* = 27	3.70 (2.85–4.63)
SM (OH) C16:1	*N* = 27	2.00 (1.41–2.39)
SM (OH) C22:1	*N* = 27	2.93 (1.49–5.19)
SM (OH) C22:2	*N* = 27	5.16 (2.66–8.19)
SM C16:0	*N* = 27	63.63 (57.34–76.77)
SM C16:1	*N* = 27	8.75 (7.02–9.81)
SM C18:0	*N* = 27	18.72 (14.45–22.25)
SM C18:1	*N* = 27	6.82 (4.94–8.54)
SM C24:0	*N* = 27	12.94 (10.32–17.38)
SM C24:1	*N* = 27	21.29 (0.52–38.43)
H1	*N* = 27	5057.30 (4151.64–5708.51)
Progesteron	*N* = 27	0.17 (0.16–0.18)
5–α–Cholestane	*N* = 27	1.79 (1.16–3.75)
Chol_Epoxide	*N* = 27	2.00 (1.88–2.13)
1, 2dilinoleoyl_PC	*N* = 27	5.47 (3.16–12.45)
1, 2dioleoyl_GLP_Na2	*N* = 27	5.38 (5.36–5.39)
D–sphingosine	*N* = 27	0.10 (0.06–0.13)
C16–CAR2	*N* = 26	0.05 (0.04–0.06)
Oleoyl–CAR	*N* = 26	0.13 (0.11–0.15)
Lanosterol	*N* = 27	134.91 (122.86–145.51)
CER_893_1	*N* = 27	1.05 (0.70–1.85)
1–palmitoyl–sn–glycero–3PC	*N* = 27	96.77 (60.97–147.00)
BSitosterol	*N* = 27	4.00 (3.06–5.24)
glyceryltrioleate1	*N* = 27	80.89 (62.60–100.17)

1, 2dioleoyl_PE	*N* = 26	1.19 (0.98–1.37)
cis–vaccenic_acid	*N* = 27	72.56 (51.36–95.72)
ArachidicAcid	*N* = 27	4.09 (3.90–4.44)
erucic acid	*N* = 27	3.01 (2.90–3.10)
StearicAcid	*N* = 27	154.65 (144.81–169.06)
PalmiticAcid	*N* = 27	15.65 (14.81–16.78)
LinoleicAcid	*N* = 27	37.61 (21.95–66.41)
DocosahexaenoicAcid	*N* = 27	5.35 (4.19–7.47)
PalmitoleicAcid	*N* = 27	31.64 (20.87–47.33)
BehenicAcid	*N* = 27	0.76 (0.47–1.23)
MyristicAcid	*N* = 27	25.08 (21.75–27.37)
LinolenicAcid	*N* = 27	2.88 (2.40–5.36)
OleicAcid	*N* = 27	119.72 (94.31–166.88)
MyristoleicAcid	*N* = 27	1.37 (0.86–1.69)
LignocericAcid	*N* = 27	4.15 (3.93–4.25)
MargaricAcid	*N* = 27	6.64 (5.36–8.14)
oleanolic acid	*N* = 27	8.46 (7.19–9.81)
tripentadecanoate TG15	*N* = 27	7.88 (6.24–10.27)
Glyceryltrilinoleate1	*N* = 27	27.80 (19.48–45.77)
glyceryltripalmitoleate1	*N* = 27	0.92 (0.56–1.21)
1linoleoyl–rac–GL	*N* = 27	2.89 (2.08–4.05)
1oleoyl_rac_GL	*N* = 27	9.61 (9.42–9.90)
1monopalmitoleoyl–rac–GL1	*N* = 27	15.18 (15.15–15.25)
desmosterol1	*N* = 27	0.47 (0.38–0.60)
CHOLESTEROL2	*N* = 26	400.71 (291.32–641.45)
CA	*N* = 27	0.19 (0.10–0.59)
CDCA	*N* = 27	0.34 (0.17–0.85)
DCA	*N* = 27	0.04 (0.02–0.31)
UDCA	*N* = 27	0.04 (0.03–0.06)
LCA	*N* = 27	0.17 (0.16–0.18)
G–CA	*N* = 27	1.63 (0.30–18.08)
G–CDCA	*N* = 27	2.09 (1.06–6.49)
G–DCA	*N* = 27	0.33 (0.11–1.08)
G–UDCA	*N* = 27	0.16 (0.11–0.35)
G–LCA	*N* = 27	0.04 (0.03–0.07)
T–CA	*N* = 27	0.47 (0.13–5.48)
T–CDCA	*N* = 27	2.39 (0.42–6.46)
T–DCA	*N* = 27	0.47 (0.14–1.42)
T–UDCA	*N* = 24	0.06 (0.02–0.25)
T–LCA	*N* = 27	0.03 (0.03–0.04)
